# Low expression of HIF1AN accompanied by less immune infiltration is associated with poor prognosis in breast cancer

**DOI:** 10.3389/fonc.2023.1080910

**Published:** 2023-02-02

**Authors:** Shasha Tang, Dongyang Liu, Yuan Fang, Liyun Yong, Yi Zhang, Mengying Guan, Xiaoyan Lin, Hui Wang, Fengfeng Cai

**Affiliations:** ^1^ Department of Breast Surgery, Tongji Hospital, School of Medicine, Tongji University, Shanghai, China; ^2^ Department of Breast Surgery, Yangpu Hospital, School of Medicine, Tongji University, Shanghai, China; ^3^ Laboratory of Tumor Molecular Biology, School of Basic Medical Sciences, Shanghai University of Medicine and Health Sciences, Shanghai, China

**Keywords:** breast cancer, hypoxia-inducible factor 1-alpha subunit inhibitor (HIF1AN), prognosis, immune infiltrating cell, biomarker

## Abstract

**Background:**

Hypoxia-inducible factor 1-alpha (HIF-1α) stability and transcriptional action are reduced by the hypoxia-inducible factor 1-alpha subunit suppressor (HIF1AN). Its inappropriate expression is associated with the development of cancer and immune control. It is yet unknown how HIF1AN, clinical outcomes, and immune involvement in breast cancer (BC) are related.

**Methods:**

Using the GEPIA, UALCAN, TIMER, Kaplan-Meier plotter, and TISIDB datasets, a thorough analysis of HIF1AN differential expression, medical prognosis, and the relationship between HIF1AN and tumor-infiltrating immune cells in BC was conducted. Quantitative real-time PCR (qRT−PCR) analysis of BC cells were used for external validation.

**Results:**

The findings revealed that, as compared to standard specimens, BC cells had significantly lower levels of HIF1AN expression. Good overall survival (OS) for BC was associated with higher HIF1AN expression. Additionally, in BC, the expression of HIF1AN was closely associated with the chemokines and immune cell infiltration, including neutrophils, macrophages, T helper cells, B cells, Tregs, monocytes, dendritic cells, and NK cells. A high correlation between HIF1AN expression and several immunological indicators of T-cell exhaustion was particularly revealed by the bioinformatic study.

**Conclusions:**

HIF1AN is a predictive indicator for breast tumors, and it is useful for predicting survival rates.

## Introduction

Breast cancer (BC) is a complicated disease with numerous classifications and exhibits both significant inter- and intra-tumor variations (1, 2). Globally, BC affects approximately 10% of women during the course of their lives (3, 4). Despite improvements in the diagnosis and treatment of BC, the management of the disease is still challenging and most patients have poor outcomes (5). ER (+) or ER (-) are the two hormone receptors used to classify BC, with the latter having fewer therapeutic decisions, especially for triple-negative breast cancers (TNBCs) (2, 6). Several studies have investigated the molecular pathways underlying the various hormonal states to reveal options for the treatment of BC.

In addition to surgery, chemo- and radiation therapy, inhibition of targeted pathways and combination immunotherapies are considered alternative treatment options (7). Given its high heterogeneity, not all BC patients benefit from immunotherapy. Researchers have demonstrated that the clinical efficacy of immunotherapy is partly influenced by the immunosuppressive tumor microenvironment (8). Therefore, specific immune-related biomarkers of BC should be explored to develop new immunotherapeutic targets and strategies to alleviate resistance.

The HIF-1A protein regulates the transcription of genes involved in response to hypoxia. The protein participates in processes the ensure the survival of cells under hypoxia (9, 10), and it has been shown that HIF1A may play a role in the development of tumor resistance to immunotherapy (11, 12). It is also involved in tumor development and metastasis (13). Hypoxia-inducible factor 1-alpha subunit suppressor (HIF1AN), also known as component suppressing HIF-1 (FIH-1), inhibits HIF-1 activity by hydroxylating the C-terminal trans-activation domain of the HIF-1α subunit, thus preventing HIF-1 from recruiting co-activators CPB/p300, which are important for the transcription of target genes (14, 15). Prior research suggests that HIF1A enhances cancer progression, spread, and metastasis by promoting angiogenesis and controlling cellular metabolism in hypoxic tumor conditions. HIF1A is upregulated in multiple malignancies and immune responses (16). Additionally, numerous studies have revealed that HIF1AN suppresses the growth of cancerous cells and might function as a potential tumor inhibitor in gastrointestinal and prostate cancer (17). The fundamental pathways *via* which HIF1AN prevents tumor growth and immunological interaction with BC are currently unknown.

To investigate the relevance of HIF1AN in BC, RNA sequencing and medical analysis process based on BC patients with identified HIF1AN collected from The Cancer Genome Atlas (TCGA) dataset. The link between HIF1AN and immunological infiltration was also investigated. This is the first in-depth investigation into the clinical, structural, and immunological features of HIF1AN gene expression.

## Materials and methods

### RNA-sequencing of patient data

Gene expression data of cases in which HIF1AN had been measured using HTSeq-FPKM or HTSeq-count, generated by the Breast invasive carcinoma (BRCA) projects, together with corresponding clinical information, were collected from the TCGA website. Normal BRCA samples and cases with an overall survival of <30 days were excluded. Level 3 HTSeq-FPKM data were transformed into transcripts per million reads (TPM) for subsequent analysis. Information from 1222 patients with BRCA was retained. Unknown or unavailable clinical data in the 1222 patients were considered to be missing values. All data used in the paper were acquired from TCGA, and hence ethics approval and informed consent were not required.

### Identification of DEGs and functional enrichment analysis

To obtain the differentially expressed genes (DEGs) for BRCA between the high and low HIF1AN expression groups, the expression profiles (HTSeq-counts) were analyzed using the DESeq2 R package (18). The threshold values used to identify DEGs were |log2FoldChange| > 1.5 and p.adj < 0.05. Then the functional enrichment analyses, including Gene Ontology (GO) functional analysis and Kyoto Encyclopedia of Gene and Genomes (KEGG) pathway were performed using the clusterProfiler package in R. The thresholds were as follows: p.adj<0.05 and q value <0.2.

### Exploration of the expression of HIF1AN and its clinical relevance

To investigate the clinical relevance of HIF1AN, we explored the university of Alabama at Birmingham cancer data analysis portal (UALCAN). UALCAN (http://ualcan.path.uab.edu) a user-friendly portal, can facilitating the analysis in various tumor sub-groups based on individual cancer stages, tumor grade, race, body weight or other clinicopathologic features (19). In our study the associations between HIF1AN expression and significant clinical characteristics, including tissue type (healthy/tumor), breast cancer subtypes, stage of cancer (stages 1, 2, 3, and 4), lymph node stage (N0, 1, 2, and 3), and cancer cluster, are investigated.

### Survival analysis of HIF1AN in breast cancer

The Kaplan-Meier plotter Database (http://kmplot.com/analysis/) was used for survival analysis (20). We used the pattern of mRNA of gene chip in Breast cancer to explore the prognostic value of HIF1AN, including overall survival (OS), recurrence-free survival (RFS), and distant metastasis-free survival (DMFS). The hazard ratio (HR) and 95% confidence interval (CI) were calculated and the differences between the survival curves were examined using log-rank tests.

### Gene set enrichment analysis

To further investigate the functions of HIF1AN in breast cancer, Gene set enrichment analysis (GSEA) was conducted using the clusterProfiler package (https://bioconductor.org/packages/release/bioc/html/clusterProfiler.html) in R (3.6.2). The low and high groups were determined according to the expression level of HIF1AN, and gene set permutations were performed 1000 times for each analysis. The low and high groups were used as the phenotype label, and gene sets with adj.p-value <0.05 and FDR q-value <0.25 were considered to be enrichment significant.

### Analysis of immune cell infiltration and its correlation with HIF1AN

The immune infiltration analysis of BRCA was performed using single-sample GSEA (ssGSEA) with the GSVA package in R (3.6.2) (https://www.bioconductor.org/packages/release/bioc/html/GSVA.html) for 24 types of immune cells in the tumor samples. Spearman correlation was applied to explore the correlations between HIF1AN and the infiltration levels of T cell exhaustion and TAM related genes, WilCoxon rank sum tests were used to reveal the association of the infiltration of immune cells with the groups with different levels of expression of HIF1AN.

### Correlation analysis between HIF1AN expression and chemokines

To further clarify the role of HIF1AN in the interaction between breast cancer and immune system, we searched the Tumor-Immune System Interactions Database (TISIDB, http://cis.hku.hk/TISIDB/index.php) (21). The relationship between HIF1AN and chemokines (such as CCL2, CXCL8, CXCL16 and CCR2) were calculated by Spearman’s correlation analysis in the database.

### Cell lines and culture

The normal breast epithelial cell line MCF10A, the human BC cell lines MCF-7, SKBR-3, and MDA-MB-453, and the Chinese Academy of Sciences’ Cell Bank of Type Culture Collection. DMEM (Gibco; Thermo Fisher Scientific, Inc.) supplemented with 10% (v/v) foetal bovine serum (Gibco; Thermo Fisher Scientific, Inc.) and a 1% (v/v) penicillin and streptomycin solution was employed to regularly cultivate SKBR-3, MCF-7, and MDA-MB-231 cells (Beyotime Institute of Biotechnology). A mammary epithelial cell environment (Procell Life Science & Technology Co., Ltd.) containing 10% horse serum, EGF, hydrocortisone, insulin, and 1% penicillin-streptomycin was employed to cultivate MCF10A cells. All cell lines were perfused employing conventional cell culture methods and grown in an incubator at 37°C with 5% CO2.

### Quantitative real-time PCR analysis

Using the RNAiso Plus Kit (cat. no. 9109; Takara Biotechnology Co., Ltd.), total RNA of the SKBR-3, MCF-7, MDA-MB-453, and MCF10A cells was extracted in accordance with the industrialist’s recommendations. Following the industrialist ‘s instructions, 1,000 ng of total mRNA were retro transcribed into cDNA employing Takara Biotechnology Co., Ltd.’s PrimeScript™ RT Reagent Kit with the Genomic DNA Eraser (cat. no. RR047). To find out if each of the target genes was expressed, TB Green Premix Ex Taq (cat. no. RR420; Takara Biotechnology Co., Ltd.) was employed in a quantitative PCR (qPCR) assay using the LightCycler^®^ 96 Instrument (Roche Diagnostics). The following primer pairs were used for qPCR: HIF1AN forward, 5’-GAGTGCCTCTACCCATACCCT-3’ and reverse, 5’-TCGTAGTCGGGATTGTCAAAGT-3’; and GAPDH forward, 5’-CATTGACCTCAACTACATGGTTT-3’ and reverse, 5’-GAAGATGGTGATGGGATTTCC-3’. qPCR was completed under the specified thermocycling situations: 95°C for 5 min, then 40 cycles of 95°C for 10 sec and 60°C for 30 sec. The proportional mRNA expression levels were normalised to those of the housekeeping gene GAPDH using the conventional 2-Cq technique, and the comparative cycle limit of the housekeeping gene GAPDH was assessed as an endogenous control. The trial was carried out three times.

### Statistical analysis

R (software v.3.6.2) was used to perform the statistical analyses. WilCoxon signed-rank tests were used to analyze the expression of HIF1AN in non-paired and paired samples. The Kaplan–Meier method was applied to survival analysis, and the differences between the survival curves were examined using log-rank tests. Correlations between HIF1AN and other genes were identified using Spearman’s correlation analysis. qRT-PCR results are presented as the mean ± standard deviation (SD) from the three independent experiments, t-test was carried out for statistical analysis with GraphPad Prism software version 7.0. A p-value <0.05 was considered as statistically significant.

## Results

### Expression of HIF1AN in various cancers and the differentially expressed genes in BC

**Figure 1:**
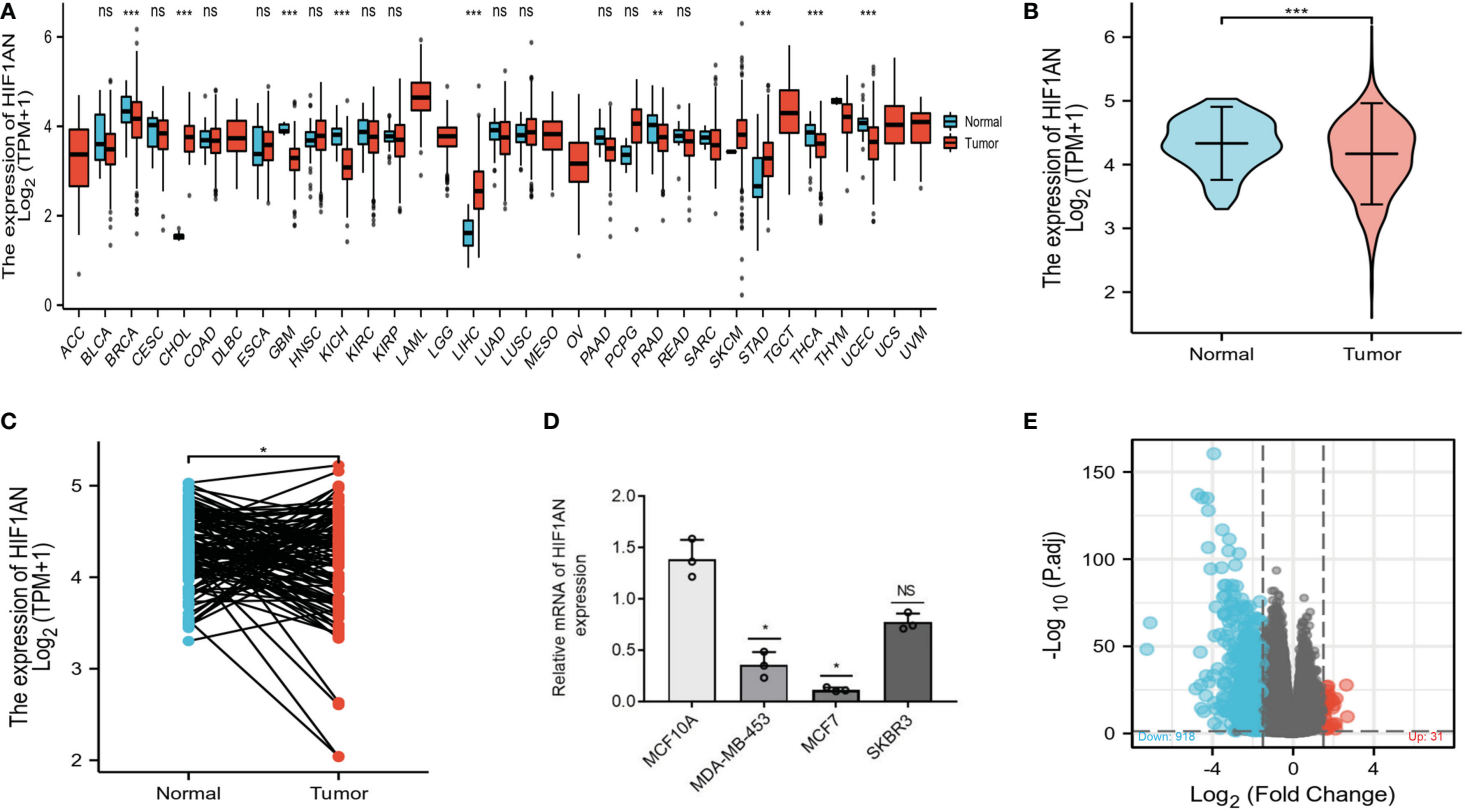
Stratified by HIF1AN levels, different mRNA expression patterns in BC patients. **(A)** The TCGA database-based expression of HIF1AN in various kinds of cancer. **(B)** According to the TCGA-BRCA data, HIF1AN expression was markedly reduced in BC cells as compared to healthy cells. **(C)** According to the TCGA-BRCA sets of data, HIF1AN expression was considerably reduced in associated BC tumour tissues as compared to neighbouring healthy tissue. ns, P ≥ 0.05; *P < 0.05; **P < 0.01; ***P < 0.001. **(D)** Evaluation of HIF1AN mRNA expression levels in breast tissues epithelium and breast cancer cell lines using reverse transcription-quantitative PCR. Mann Whitney U analysis of the relationship between TCGA BRCA datasets’ HIF1AN gene expression; **(E)** High- and low-HIF1AN expression cohorts’ mRNA expression patterns are displayed. Volcano graphs are used to visualize information.

The differential expression of HIF1AN between various cancers and nearby healthy tissue was assessed on the TCGA database. As shown in **Figure 1A**, the expression of HIF1AN was downregulated in most cancers such as breast cancer (BRCA), thyroid cancer (THCA), prostate adenocarcinoma (PRAD), and uterine corpus endometrial carcinoma (UCEC). It expression was in stomach adenocarcinoma (STAD), cholangiocarcinoma (CHO) and liver hepatocellular (LIHC).

To further verify the findings for BC, 1222 BC samples from the TCGA database were examined. HIF1AN expression levels were lower in BC (1109 samples) than in normal tissues (113 samples) (**Figure 1B**). Moreover, HIF1AN expression was lower than in matched adjacent normal tissue (**Figure 1C**). According to RT-qPCR analysis, the expression level of HIF1AN in all three types of BC cells (SKBR-3, MCF-7, MDA-MB-453) was significantly lower compared with that of MCF10A cells, which is consistent with the aforementioned results (**Figure 1D**). The 1222 BC patients were divided into two cohorts, elevated and low HIF1AN expression cohorts, depending on the median HIF1AN expression in BC tumors. The mRNA expression levels of the two cohorts were compared. In the elevated HIF1AN cohort, 949 mRNAs, comprising 31 elevated and 918 reduced genes, were identified as DEGs (absolute value of fold change >1.5, P < 0.05) (**Figure 1E**).

### Association of HIF1AN expression with clinicopathological features in BC individuals

**Figure 2:**
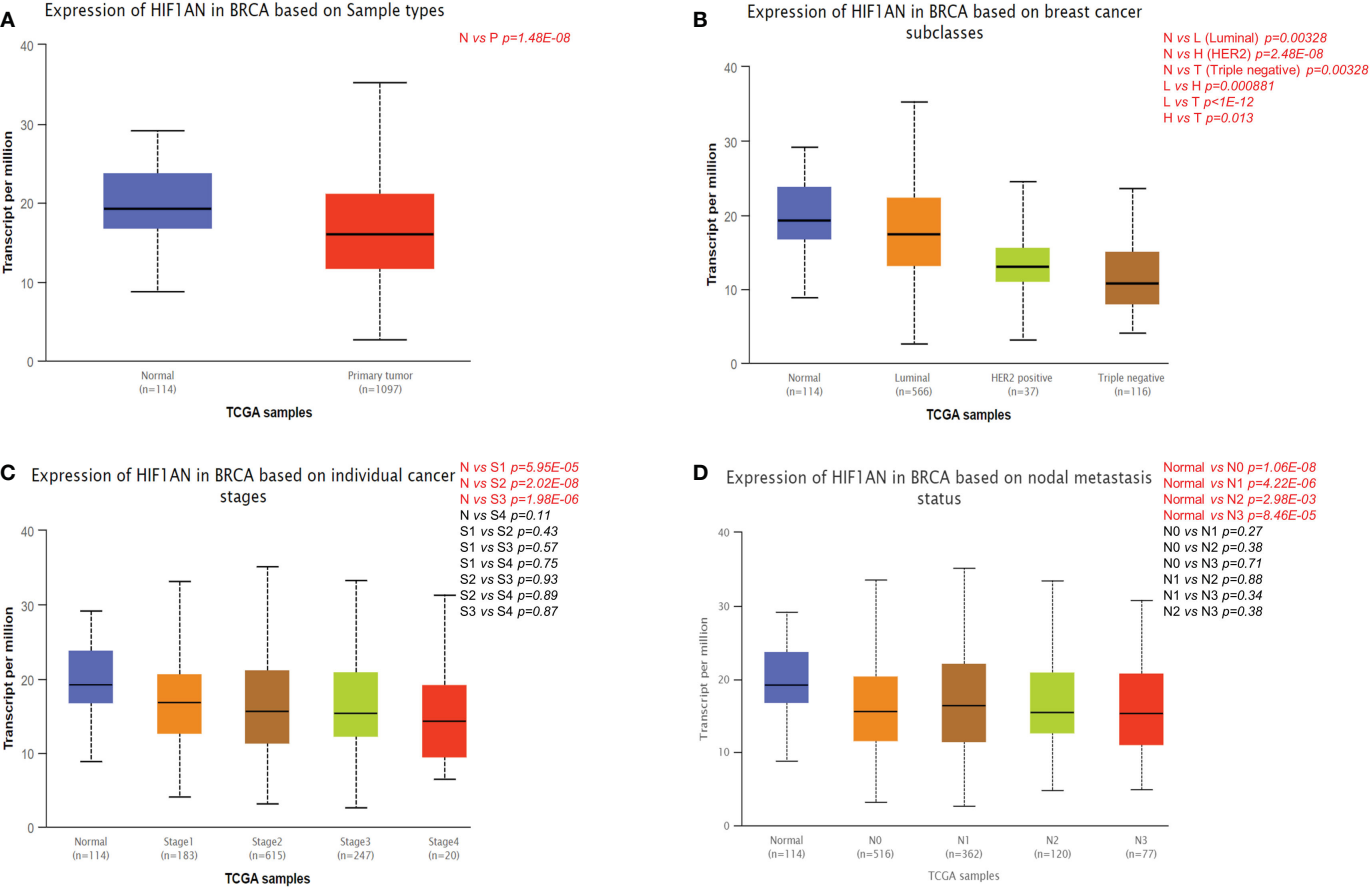
Correlation between HIF1AN expression level and clinicopathological variables of BC through the UALCAN datasets. **(A)** HIF1AN expression was remarkably downregulated in breast primary tumor than that in normal tissues. **(B)** HIF1AN expression was lower in luminal, HER2 positive and triple negative BC than in normal tissues. **(C)** HIF1AN expression in middle-stage and late-stage BC was substantially lower than in the early phase. **(D)** HIF1AN expression at all stages of lymph node stage specimens was substantially lower in BC than in healthy one; P, primary tumor; S1, stage 1; S2, stage 2; S3, stage 3; S4, stage 4.

The differential expression of HIF1AN in BC and healthy samples was explored by UALCAN as shown in **Figure 2A**. Compared to normal cells, the expression of HIF1AN was markedly significantly inhibited in BC cells (**Figure 2A**). For cancer patients with HIF1AN expression, the number of clinical and pathological factors, molecular subtypes, tumor phases (phase 1, 2, 3, and 4), and lymph node phase (N0, 1, 2 and 3) were examined. **Figure 2B** shows that compared to healthy tissues, the expression of HIF1AN was decreased in Luminal, HER2 positive, and triple negative BC. Furthermore, the expression of HIF1AN was decreased as the tumor level increased. Notably, middle-stage and late-stage BC had much lower expression of HIF1AN than early-stage BC (**Figure 2C**). HIF1AN expression was significantly decreased in BC than in all phases of lymph node phase specimens (**Figure 2D**). These findings suggest that the level of cancer is related to reduced HIF1AN.

### Decreased HIF1AN is linked to poor survival in breast cancer patients

**Figure 3:**
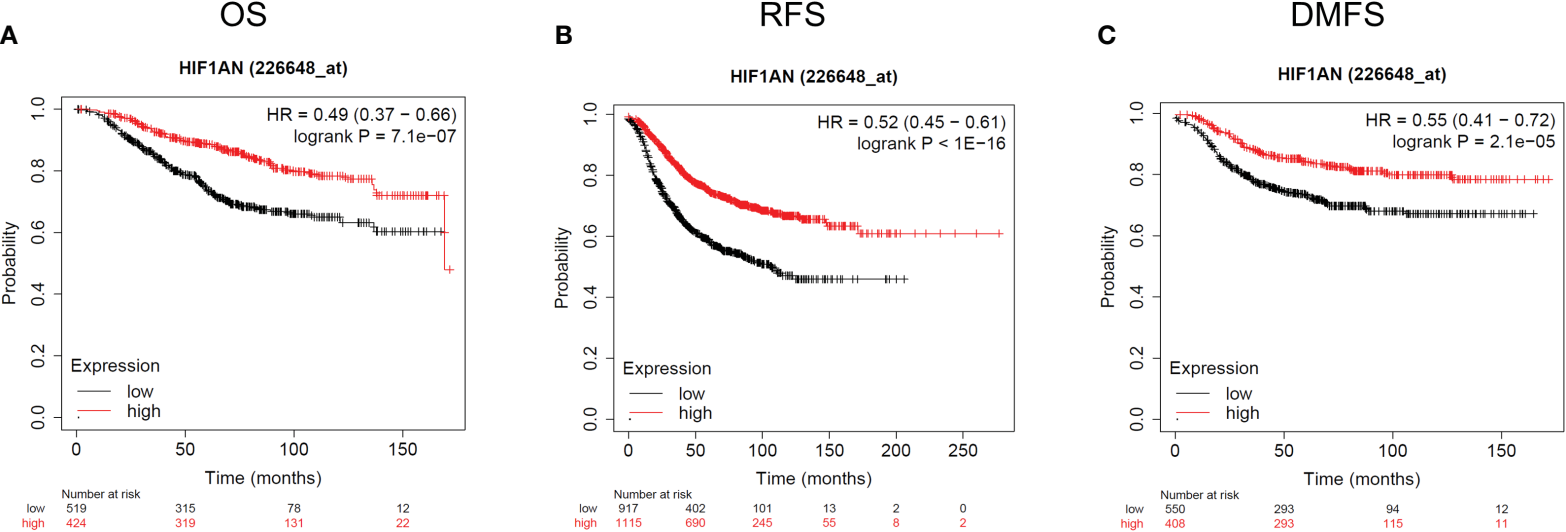
Kaplan-Meier survival curves comparing the elevated and reduced expression of HIF1AN in BC. Survival curves of OS **(A)**, RFS **(B)**, and DMFS **(C)**. OS, overall survival; RFS, recurrence-free survival, DMFS, distant metastasis-free survival.

The expression of HIF1AN in BC patients was lower than in healthy individuals. Therefore, there is a need to further investigate the relationship between HIF1AN and tumor rates. To assess the relationship between HIF1AN and prognostic outcomes in BC, KM survival curves were to explore the connection between HIF1AN and illness prognosis. In the group 226648-at, it is noteworthy that higher levels of HIF1AN expression were associated with better outcomes for BC (overall survival (OS): HR = 0.49, p < 0.001; recurrence-free survival (RFS): HR = 0.52, p < 0.001; distant metastasis-free survival (DMFS): HR = 0.55, p <0.001) (**Figure 3**).

Next, using the PAM50 subtype approach, we evaluated the likelihood that HIF1AN expression would be present in various subtypes. The healthy breast-like subtype group showed better OS when HIF1AN was highly expressed (p=0.027). The luminalA, luminalB, and basal-like subtype groups, although, did not show any major changes, while the HER2 subtype cohort showed a tendency in the other direction (p=0.027). These findings imply that the outcome of various BC subtypes is correlated with the expression level of HIF1AN.

### Predicted biological function and pathways of HIF1AN in BC

**Figure 4:**
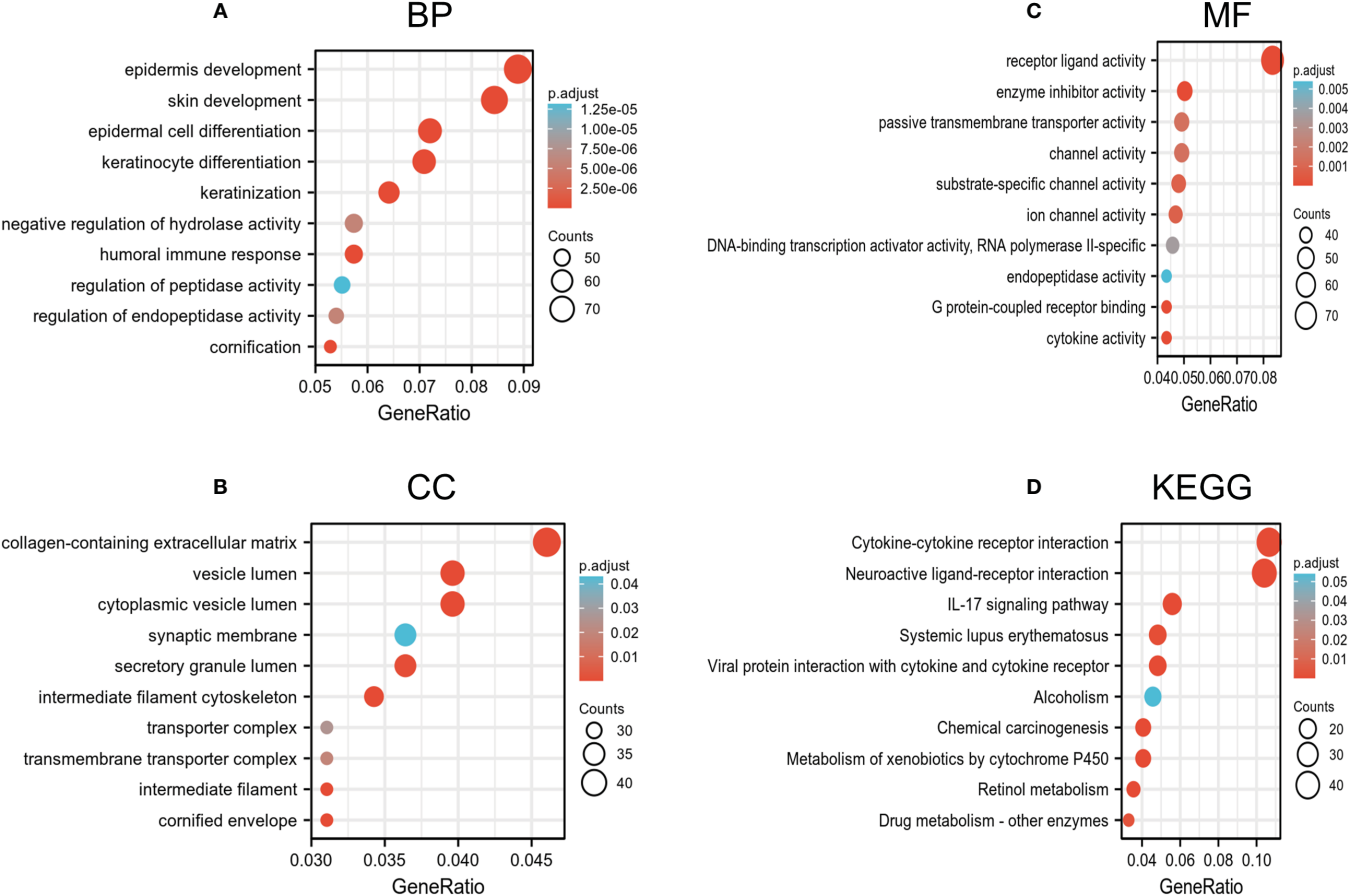
Go and KEGG enrichment test of genes associated with HIF1AN in BC cells in the TCGA-BRCA data. **(A–C)** Go enrichment evaluation revealed the BP (biological processes), CC (cellular components), and MF (molecular function) of co-expressed genes with HIF1AN. **(D)** substantially enriched KEGG terms derived from KEGG enrichment test of co-expressed genes with HIF1AN.

Genes co-expressed with HIF1AN (|logFC| > 1, P.adj <0.05) were chosen to perform gene analyses once their bioactivity was determined. Epidermis progression, skin growth, epidermal cell differentiation, and keratinocyte differentiation were all considerably elevated in GO terms used to describe biological processes (BP) (**Figure 4A**). For the cellular component, extracellular matrix, vesicle lumen, and cytoplasmic vesicles that hold collagen were enriched (CC) (**Figure 4B**). Receptor-ligand action, enzyme suppressing activity, and channel activity were all highly enriched, according to the molecular function (MF) study (**Figure 4C**). Additionally, KEGG results suggests that the IL-17 signaling mechanism, neuroactive ligand-receptor activity, and cytokine-cytokine receptor interplay were dominant processes (**Figure 4D**). Generally, the findings suggested that HIF1AN and the genes its co-expresses may be involved in cell signaling, which may control BC’s biological pathways.

**Figure 5:**
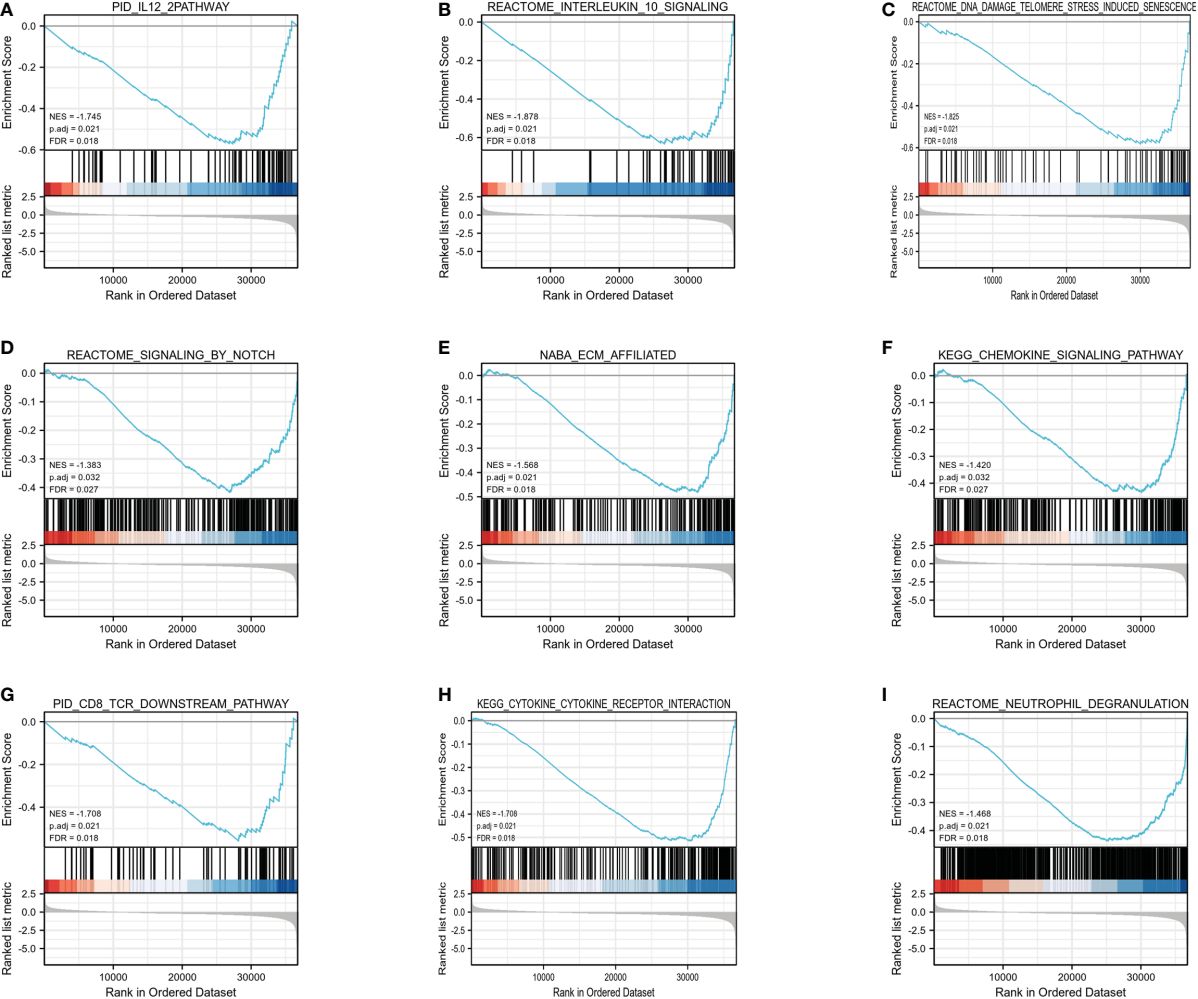
Enrichment plots from the gene set enrichment analysis (GSEA). **(A)** IL12 mechanism, **(B)** interleukin10 signaling, **(C)** DNA damage telomere stress induced senescence, **(D)** Notch signaling, **(E)** ECM affiliated, **(F)** chemokine signaling pathway, **(G)** CD8 TCR downstream pathway, **(H)** cytokine-cytokine receptor interaction, and **(I)** neutrophil degranulation were substantially enriched in HIF1AN-associated BC. NES, normalized enrichment scores; FDR, false discovery rate.

Furthermore, GSEA was carried out using the normalized enrichment score (NES) and FDR (false discovery rate) q-value to clarify the potential biological mechanisms controlled by HIF1AN between elevated and reduced HIF1AN expression cohorts. As illustrated in **Figure 5**, a number of signal mechanisms, such as Notch signaling, cell-surface contacts, CD8 TCR downstream pathway, and chemokine signaling pathway, were substantially concentrated in the cohort with decreased HIF1AN expression.

### Correlation of HIF1AN expression and immunity cells infiltration in BC

**Figure 6:**
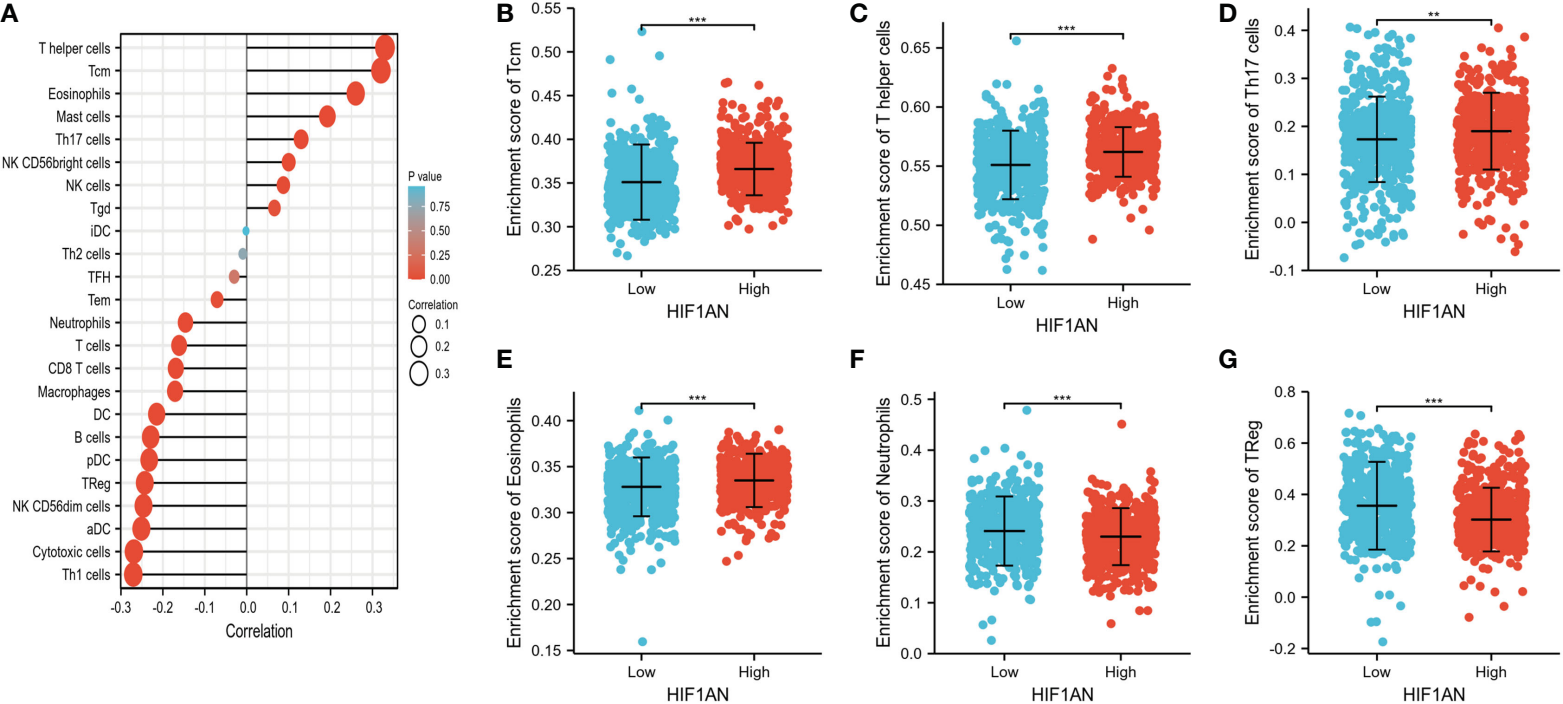
Correlation of immune cell infiltration and HIF1AN expression in BC females. **(A)** correlations among infiltration levels of 24 kinds of immune cell and HIF1AN expression profiles by Spearman’s evaluation test. Illustrated is the comparison of infiltration levels of most correlated immune cells, containing Tcm **(B)**, T helper cells **(C)**, Th17 cells **(D)**, Eosinophils **(E)**, Neutrophils **(F)** and Treg **(G)** between high and low HIF1AN expression groups. DCs, dendritic cells; aDCs, activated DCs; iDCs, immature DCs; pDCs, plasmacytoid DCs; Th, T helper cells; Th1, type 1 Th cells; Th2, type 2 Th cells; Th17, type 17 Th cells; Treg, regulatory T cells; Tgd, T gamma delta; Tcm, T central memory; Tem, T effector memory; Tfh, T follicular helper; NK, natural killer; ns: P ≥ 0.05, *P < 0.05, ** P < 0.01, and *** P < 0.001.

One of the main elements influencing tumor growth is immune infiltration. Notably, 24 different types of immune cells were found in breast tumor using ssGSEA. The relationship between immune cell infiltration and HIF1AN expression was then examined employing Spearman’s analysis. **Figure 6A** reveals significant positive correlation between HIF1AN expression and Tcm cells (R = 0.320, P <0.001), eosinophils (R = 0.260, P <0.001), T helper cells (R = 0.330, P <0.001), and NK cells (R = 0.087, P = 0.004). However, there was a negative connection between HIF1AN and macrophages (R = -0.171, P <0.001), neutrophils (R = -0.146, <P 0.001), Th1 cells (R = -0.271, P <0.001), CD8 T cells (R = -0.169, P <0.001), and aDC cells (R = -0.251, P <0.001). Moreover, the rates of immune cell infiltration in various HIF1AN cohorts were assessed, including Tcm cells (**Figure 6B**), T helper cells (**Figure 6C**), Th17 cells (**Figure 6D**), eosinophils (**Figure 6E**), neutrophils (**Figure 6F**), and Treg cells (**Figure 6G**). The findings were in line with those shown in **Figure 3A**, demonstrating the significance of HIF1AN in immune infiltration of Breast malignancy.

The relationship between HIF1AN and several TIL indicators (neutrophils, T cells and associated variants, CD8+/CD4+ T cells, NK cells, B cells, monocytes, DCs, TAMs, M1 macrophages, and M2 macrophages) in BC was investigated using the GEPIA and TIMER datasets. It was found that most TIL identifiers were correlated with HIF1AN. Additionally, several functional T cells, particularly Tregs, Th1, Th2, Th17, Tfh cells, and fatigued T cells, were examined. Results showed that HIF1AN was particularly strongly correlated with TILs (**Supplementary Table 1**).

**Figure 7:**
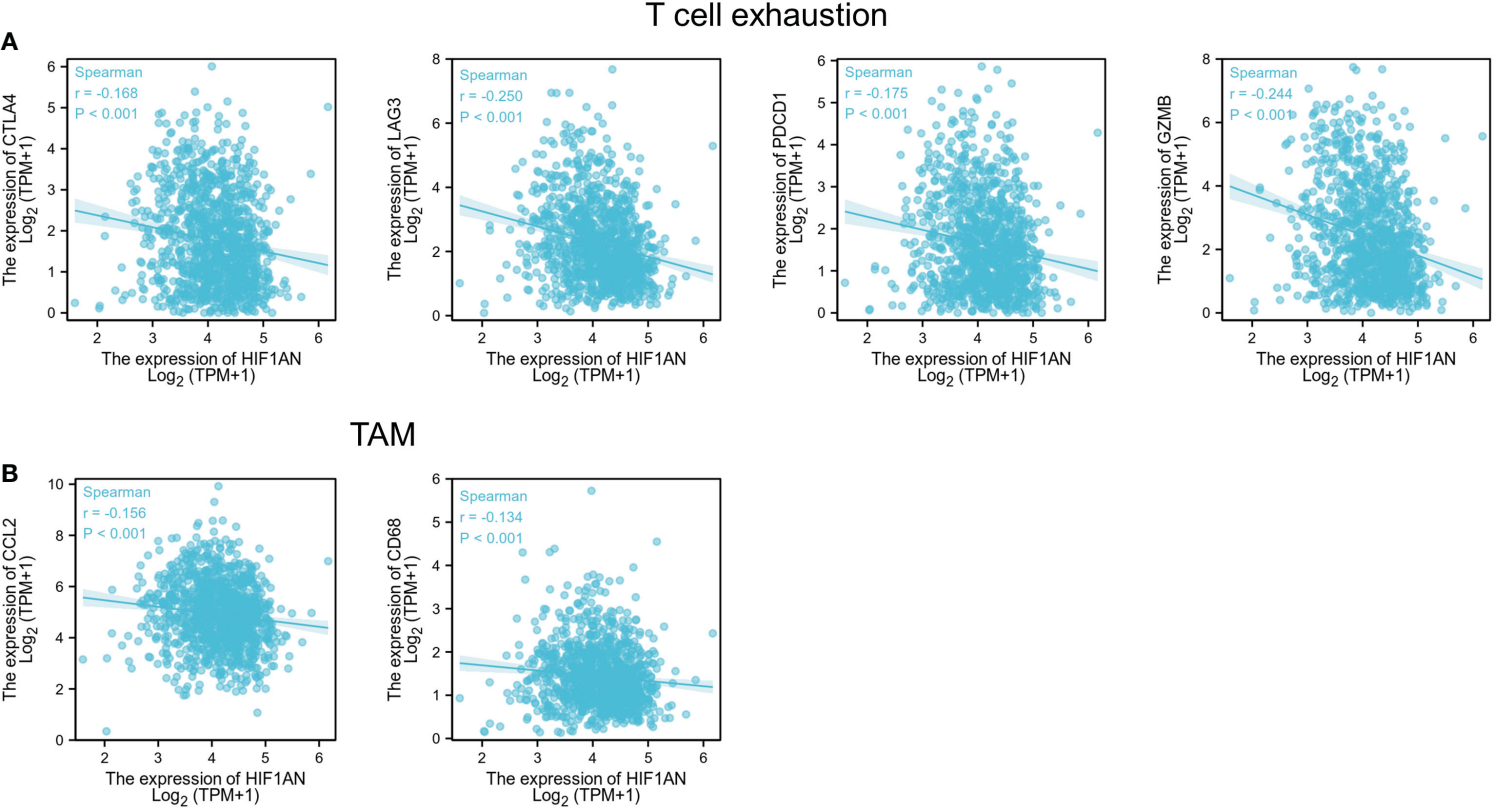
HIF1AN expression correlated with T cell exhaustion and markers include CCL2 and CD68 of TAMs in BC; Scatterplots of correlations between HIF1AN expression and gene indicators of **(A)** T cell exhaustion, **(B)** TAMs.

Interestingly, the findings suggested a correlation between HIF1AN in breast malignancy and PDCD1, LAG3, CTLA4, and GZMB of T cell exhaustion as well as chemokine ligand CCL2 of TAMs (**Figures 7A, B**). proving that HIF1AN may have a role in controlling T cell fatigue in breast tumors.

### Correlation between the HIF1AN and chemokines in BC patients

Chemokines regulate infiltration of immune cells (22). Here, we found that HIF1AN expression was correlated with chemokines. Particularly, HIF1AN expression was significantly (p < 0.001) linked to CCL2 (Cor = -0.338), CCL3 (Cor = -0.222), CCL4 (Cor = -0.303), CCL5 (Cor = -0.367), CCL8 (Cor = -0.264), CCL11 (Cor = -0.164), CCL13 (Cor = -0.297), CCL7 (Cor = -0.279), CCL17 (Cor = -0.264), CCL19 (Cor = -0.221), CX3CL1 (Cor = -0.351), CXCL9 (Cor = -0.234), CXCL10 (Cor = -0.282), CXCL13 (Cor = -0.21), and XCL2 (Cor =- 0.286). Furthermore, HIF1AN expression was also related with chemokine receptors (p < 0.001), including CCR1 (Cor = -0.203), CCR2 (Cor = -0.151), CCR5 (Cor = -0.179), CCR7 (Cor = -0.223), CCR10 (Cor = -0.275), CXCR3 (Cor = -0.285), CXCR4 (Cor = -0.273), CXCR5 (Cor = -0.211), CXCR6 (Cor = -0.26) and CX3CR1 (Cor = 0.187) (**Table 1**). The above outcomes further proved that HIF1AN may modulate in breast malignancy.

**Table 1:** Correlation analysis between the expression of HIF1AN and Chemokines & Receptors in breast cancer at TISIDB datasets.

Chemokines & Receptors	HIF1AN	
	Cor	*p* Value
CCL2	-0.338	<0.001
CCL3	-0.222	<0.001
CCL4	-0.303	<0.001
CCL5	-0.367	<0.001
CCL7	-0.279	<0.001
CCL8	-0.264	<0.001
CCL11	-0.164	<0.001
CCL13	-0.297	<0.001
CCL17	-0.264	<0.001
CCL18	-0.264	<0.001
CCL19	-0.221	<0.001
CXCL8	-0.19	<0.001
CXCL11	-0.266	<0.001
CXCL13	-0.21	<0.001
CXCL16	-0.324	<0.001
XCL1	-0.27	<0.001
XCL2	-0.286	<0.001
CCR1	-0.203	<0.001
CCR2	-0.151	<0.001
CCR5	-0.179	<0.001
CCR7	-0.223	<0.001
CCR10	-0.275	<0.001
CXCR3	-0.285	<0.001
CXCR4	-0.273	<0.001
CXCR5	-0.211	<0.001
CXCR6	-0.26	<0.001
CX3CR1	0.187	<0.001

## Discussion

Hypoxic stress is a characteristic of most solid tumors and is associated with adverse clinical outcomes (23). HIF1A is the main regulator of hypoxic response. Recent studies have demonstrated that the tumor immune escape is closely related to epithelial-mesenchymal transformation (EMT) of tumor cells and regulates the tumor microenvironment (24). In particular, HIF1A is stimulates the EMT in cancer cells, and modifies the immune function of tumor cells, and promotes immune escape (16). HIF1AN, an asparagine hydroxylase and an upstream regulation gene of HIF1A, acts as an inhibitor of HIF1A and is an important transcription factor that affects responses to hypoxia (17). Under normoxic conditions, HIF1AN inhibits the transcriptional activity of HIF1A, to prevent the transcription of the HIF1A-mediated gene. However, under hypoxic conditions, the inhibition is relieved, allowing HIF1A to recruit CBP/p300, resulting in expression of the target gene (25). Although HIF1AN has been well investigated in several kinds of malignant tumors, its clinical significance and possible regulatory role in immunity in breast malignancy is unknown.

In the current work, a bioinformatics analyses were done to examine the bioactivities of HIF1AN in BC. According to our studies, HIF1AN expression was downregulated in breast cancer females which correlated with poor prognosis (**Figure 3**). These findings further showed that decreased HIF1AN expression was directly linked to the level of immune cell, immunostimulator, immunological inhibitor, receptor, and chemokine infiltration in BC (**Figures 6**, **7**, **Table 1**). Therefore, we concluded that HIF1AN may be a tumor suppressor with the potential to be a treatment target in women with breast cancer. It is also likely to be an indicator of immune infiltration in BC.

The expression of HIF1AN in BC was determined using a separate database. In normal cells and cancer cells, HIF1AN was considerably reduced in BC cells, and it was related to the tissue level (**Figure 2**). Furthermore, low expression of HIF1AN was correlated with negative clinical outcomes (**Figure 3**). These findings imply that HIF1AN may act as a tumor inhibitor in BC and slow the spread of breast cancer. Earlier studies also showed that HIF1AN deficiency increased VEGF expression in head and neck cancer, elevated the expression of HIF1AN to suppress the oncogenic progression of head and neck squamous cell carcinoma (26). Inhibition of HIF1Awas found to be a therapeutic strategy for the human colorectal cancer (17). Furthermore, a comparable investigation revealed that miR-135b-5p may promote the growth of ovarian cancer cells by suppressing HIF1AN the expression (27). These studies showed the cancer-inhibitory role of HIF1AN in other tumors, which was similar to the results of our analysis in BC.

Surprisingly, the PAM50 test showed that the effect of HIF1AN expression on survival rates varied across different breast cancer subtypes. Decreased HIF1AN expression, particularly in the normal-like subtype, was associated with poor prognosis but opposite results were obtained in the HER2 subtype. These findings imply that HIF1AN has diverse functions in different BC types, but it is important to take into account sampling errors in the five BC subtypes.

TCGA datasets were subjected to GO and KEGG analyses of the HIF1AN-coexpressed genes and GSEA analyses of HIF1AN to better investigate the cellular roles and related concepts of HIF1AN in BC. Results of GO analysis revealed physiological systems associated with the formation of the epidermis, the extracellular structure that contains collagen, and receptor-ligand activity (**Figure 4**). The KEGG analyses revealed two major systems: cytokine-cytokine receptor interaction and neuroactive ligand-receptor relationship (**Figure 5**). A previous study showed that carcinogenesis and progression were thought to be influenced by neuroactive ligand-receptor interaction in several malignant forms, including glioma (25), renal cell carcinoma (28), colorectal cancer and hepatocellular carcinoma (29). HIF1AN might contribute to the neuroactive ligand-receptor interaction and cell signaling processes needed for BC to start and spread malignancy.

In the low HIF1AN expression phenotype, GSEA findings indicate that a number of routes were considerably dominated, including the chemokine signaling pathway, CD8 TCR downstream mechanism, interleukin 10 and ECM affiliated signaling (**Figure 4**). These mechanisms show a tight connection with cancer or the inflammatory reaction. Increased HIF1AN expression was associated with chronic colitis in a previous study (30).

To explore the immune infiltration status of BC and its association with HIF1AN, we assessed immune cell populations and their correlation with HIF1AN expression levels. Our results illustrated that HIF1AN expression is negatively correlated with numerous immune cells. High HIF1AN levels are associated with decreased infiltration of several immunocellular markers including CD8 T cells, B cells, DC cells and neutrophils (**Figure 6**, **7** and **supplementary materials**). These results need to be further validated through *in vitro* and *in vivo* experiments. Nevertheless, they suggest that the role of HIF1AN in TILs attraction and cancer microenvironment through which immune cells regulate tumorigenesis, cancer development and metastasis, as well as affect the efficacy and/or resistance to chem- and immunotherapy (31). Immunosuppressive cells like Treg cells and neutrophils can inhibit antitumor response and high levels of these cell types in a hypoxic environment significantly modulate the immune microenvironment (32). In the present study, we observed a similar effect, with higher levels of Tregs and neutrophil cells found in BC females with low HIF1AN expression than in patients with elevated HIF1AN expression. This indicates that HIF1AN may be a favorable factor that modulate the immune microenvironment of BC patients.

Most studies have shown that immune checkpoints have cancer immunosuppressive effects and are the primary immunotherapeutic strategy (32). To date, multiple clinical studies have demonstrated the efficacy of immunosuppressants against PD1/PDL1 in various malignancies, including TNBC (33). However, resistance to immune therapy limits their clinical application (34). Therefore, improving malignant tissue response to immune checkpoint suppressors and cytokines has a significant impact in cancer treatment. It was demonstrated that low oxygen increased PD-L1 expression on macrophages in the tumor milieu (35). Additionally, it was shown that hypoxia significantly reduced the ability of CTLs to kill cancer cells (36). This is likely because HIF1A affects the sensitivity of malignant cells to CTL-mediated killing by increasing expression of NANOG and microRNA (miR)-210 (37, 38). Additionally, combining anti-PD-1 with reducing HIF1A concentrations by pharmacologically inhibiting Axl decreases the main tumour and metastatic loads in a preclinical model of HER2+ breast cancer, indicating a viable treatment strategy in BC (39). According to our findings, PD1, LAG3, CTLA4, and GzmB were all inversely correlated with T cell fatigue, as were elevated doses of HIF1AN expression. GzmB should be considered a sign of late T cell depletion. The exhausted T cells showed a diminished function in a hierarchical way (40). It is suggested that lower expression of HIF1AN is correlated with a higher level of T cell exhaustion markers, which indicates that the tumor may under a hypoxia state, the HIF1A is activated, and the T cells enter a cellular state called “Exhaustion”, they unable to clear the tumor cells. Therefore, decrease HIF1A activity by HIF1AN inhibition could provide an antitumorigenic microenvironment. Additionally, GzmB is an indicator for NK cell (Natural Killer)-mediated killing. One of the main methods by which NK cells destroy tumor cells is by producing cytotoxic granules containing perforin (PRF1) and GzmB. According to reports, lack of oxygen negatively affects NK-mediated killing in addition to impairing CTL-mediated death. Evidence indicates that ischemic cells preferentially activate phagocytosis to destroy the proapoptotic protein GzmB, which prevents the NK system from destroying cancer cells (41). Our findings, therefore, indicated that there is a bad correlation between GzmB expression and HIF1AN. This suggested that lack of oxygen cells may correspond with greater NK cellular function rather than a suppressive impact in reduced HIF1AN conditions.

Additionally, this study found that high expression level of HIF1AN were also negatively correlated with chemokines and receptors (**Table 1**). Most tumors produce two types of chemokines, CXC and CC. Studies have shown that CCL5 promotes tumor cell growth and inhibits paracrine and autocrine apoptosis of breast cancer (42). This indicate that decreased HIF1AN level may through chemokines regulate the tumor growth and apoptosis. Other studies have reported that HIF induced the release of proinflammatory and proangiogenic substances by breast cancer cells, adipocytes, infiltrating CD8^+^ T cells, and other stromal cells, suggesting an intricate interplay between HIFs, proinflammatory factors derived from tumor and various TME cells, and angiogenesis that has yet to be fully elucidated (43, 44), and HIF1AN may also regulate angiogenesis and tumor microenvironment through chemokines and cytokines. Furthermore, chemokines and cytokines play an essential role in leukocyte recruitment. These results reflected that higher HIF1AN may correlated with lower level of immune response in the tumor microenvironment, and higher HIF1AN may also correlated with lower level of angiogenesis and proliferation, which indicate a better situation of the patient.

In conclusion, this study demonstrated that high HIF1AN expression may be associated with a favorable prognosis of BC patients. HIF1AN was also found to be involved in immune infiltration mechanisms, to modulate the tumor immune microenvironment. Further *in vitro* and *in vivo* investigation and validation experiments are necessary to confirm these observations. This study is the first systematic in-clinic investigation of core correlations of HIF1AN in BC, exploring its potential molecular mechanism and function in modulating the tumor microenvironment. Thus, HIF1AN is a potential prognostic factor and therapeutic target of BC.

## Data availability statement

The datasets presented in this study can be found in online repositories. The names of the repository/repositories and accession number(s) can be found in the article/**Supplementary Material**.

## Author contributions

FC and HW designed and managed the entire study; ST, DL, YF and LY collected and analyzed the data; ST and DL wrote the main manuscript text; LY, YZ, MG and XL performed the generated figures. HW assisted with the article revision. All authors contributed to the article and approved the submitted version.
