# Supplementary figures and images for: Low expression of HIF1AN accompanied by less immune infiltration is associated with poor prognosis in breast cancer
Source: Front Oncol. 2023 Feb 2;13:1080910. doi: 10.3389/fonc.2023.1080910 (PMC9932925; doi:10.3389/fonc.2023.1080910)

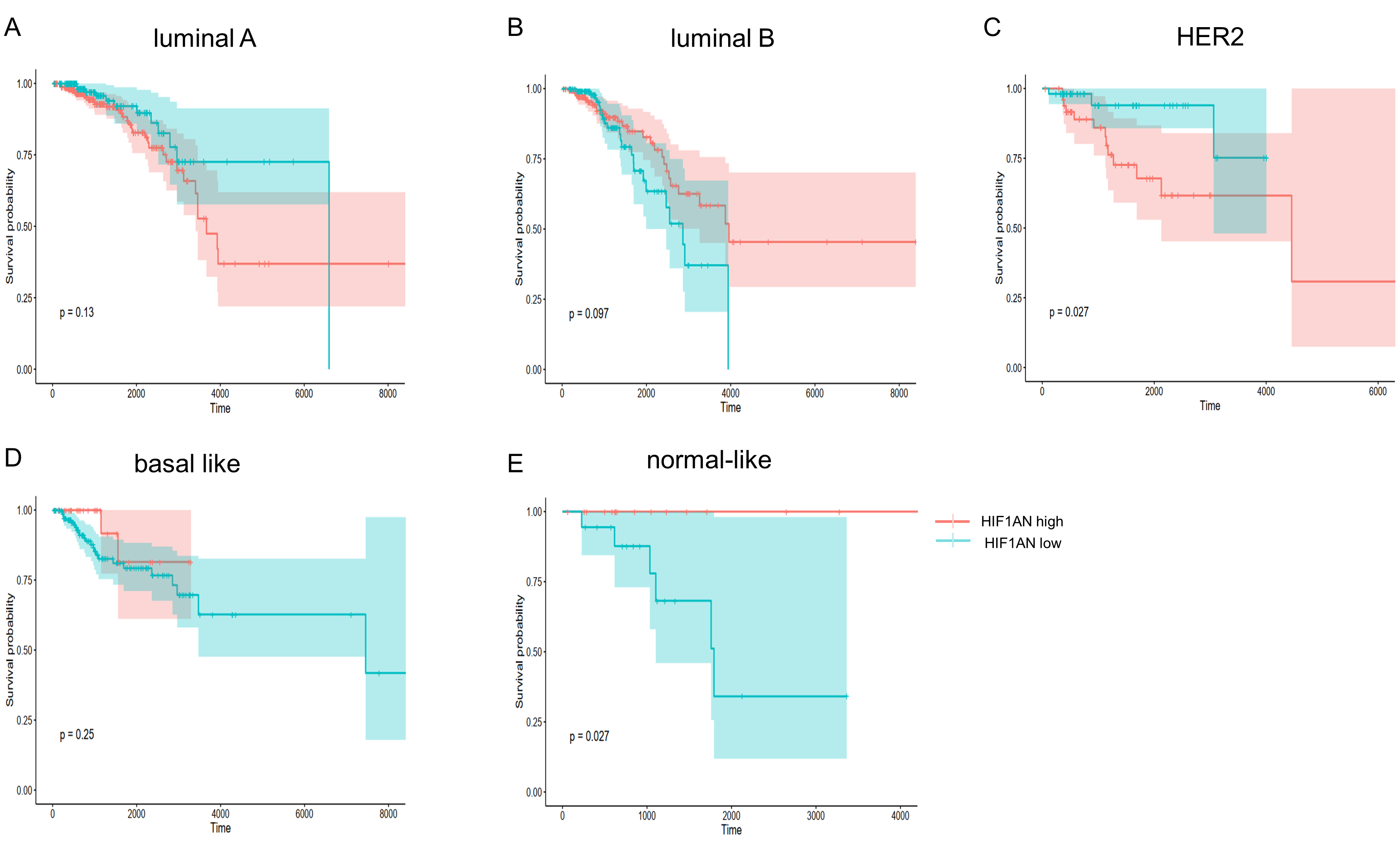

Supplement: Supplementary file 1 [file Image_1.tif]
