# Supplementary material for: Low expression of HIF1AN accompanied by less immune infiltration is associated with poor prognosis in breast cancer
Source: Front Oncol. 2023 Feb 2;13:1080910. doi: 10.3389/fonc.2023.1080910 (PMC9932925; doi:10.3389/fonc.2023.1080910)
Supplement: Supplementary file 2 [file Table_1.docx]

**Supplementary Table 1** Correlation analysis between HIF1AN and related genes and markers of immune cells.

| Description | Gene markers | None |  | Purity |  |  |
| --- | --- | --- | --- | --- | --- | --- |
|  |  | Correlation | P | Correlation | P |  |
| CD8+T cell | CD8A | -0.051 | ns | -0.008 | ns |  |
|  | CD8B | -0.146 | **** | -0.118 | *** |  |
| T cell (general) | CD3D | -0.146 | **** | -0.114 | *** |  |
|  | CD3E | -0.095 | * | -0.005 | ns |  |
|  | CD2 | -0.066 | * | -0.021 | ns |  |
| B cell | CD19 | -0.135 | **** | -0.106 | *** |  |
|  | CD79A | -0.143 | **** | -0.115 | *** |  |
| Monocyte | CD86 | 0.017 | ns | 0.057 | ns |  |
|  | CD115 (CSF1R) | 0.112 | *** | -0.044 | *** |  |
| TAM | CCL2 | -0.136 | **** | -0.391 | *** |  |
|  | CD68 | 0.05 | ns | 0.03 | ns |  |
|  | IL10 | 0.034 | ns | 0.042 | ns |  |
| M1 macrophage | INOS (NOS2) | 0.03 | ns | 0.044 | ns |  |
|  | IRF5 | -0.001 | ns | 0.011 | ns |  |
|  | COX2 (PTGS2) | -0.059 | ns | -0.025 | ns |  |
| M2 macrophage | CD163 | 0.082 | ** | 0.116 | **** |  |
|  | VSIG4 | 0.093 | ** | 0.125 | **** |  |
|  | MS4A4A | 0.029 | ns | 0.073 | * |  |
| Neutrophils | CD66b (CEACAM8) | -0.044 | ns | -0.05 | ns |  |
|  | CD11b (ITGAM) | 0.182 | **** | 0.22 | **** |  |
|  | CCR7 | -0.036 | ns | 0.016 | ns |  |
| Natural killer cell | KIR2DL1 | -0.071 | * | -0.055 | ns |  |
|  | KIR2DL3 | -0.057 | ns | -0.027 | ns |  |
|  | KIR2DL4 | -0.161 | **** | -0.131 | **** |  |
|  | KIR3DL1 | -0.077 | * | -0.049 | ns |  |
|  | KIR3DL2 | -0.13 | **** | -0.101 | ** |  |
|  | KIR3DL3 | -0.094 | * | -0.092 | ** |  |
|  | KIR2DS4 | -0.139 | **** | -0.106 | *** |  |
| Dendritic cell | HLA-DPB1 | -0.061 | * | -0.025 | ns |  |
|  | HLA-DQB1 | -0.072 | ns | -0.05 | ns |  |
|  | HLA-DRA | 0.035 | ns | 0.087 | ** |  |
|  | HLA-DPA1 | 0.06 | * | 0.115 | *** |  |
|  | BDCA-1 (CD1C) | 0.067 | * | 0.137 | **** |  |
|  | BDCA-4 (NRP1) | 0.235 | **** | 0.271 | **** |  |
|  | CD11c (ITGAX) | 0.038 | ns | 0.094 | ** |  |
| Th1 | T-bet (TBX21) | -0.118 | **** | -0.084 | ** |  |
|  | STAT4 | 0.009 | ns | 0.062 | * |  |
|  | STAT1 | 0.147 | **** | 0.161 | **** |  |
|  | IFN-γ (IFNG) | -0.121 | **** | -0.09 | ** |  |
|  | TNF-α（TNF) | 0.028 | ns | 0.065 | * |  |
| Th2 | GATA3 | 0.475 | **** | 0.4666 | **** |  |
|  | STAT6 | 0.387 | **** | 0.375 | **** |  |
|  | STAT5A | 0.158 | **** | 0.183 | **** |  |
|  | IL13 | -0.076 | ns | -0.043 | ns |  |
| Tfh | BCL6 | 0.27 | **** | 0.309 | **** |  |
|  | IL21 | -0.024 | ns | -0.003 | ns |  |
| Th17 | STAT3 | 0.474 | **** | 0.477 | **** |  |
|  | IL17A | -0.094 | ** | -0.103 | * |  |
| Treg | FOXP3 | -0.042 | ns | 0.014 | ns |  |
|  | CCR8 | 0.135 | **** | 0.177 | **** |  |
|  | STAT5B | 0.441 | **** | 0.452 | **** |  |
|  | TGFβ (TGFB1) | 0.088 | ** | 0.128 | **** |  |
| T cell exhaustion | PD-1 (PDCD1) | -0.191 | **** | -0.167 | **** |  |
|  | PDL1 (PDCD1LG2) | 0.014 | * | 0.069 | * |  |
|  | CTLA4 | -0.16 | **** | -0.129 | **** |  |
|  | LAG3 | -0.261 | **** | -0.241 | **** |  |
|  | TIM-3 (HAVCR2) | 0.084 | ** | 0.12 | *** |  |
|  | GZMB | -0.223 | **** | -0.242 | **** |  |

TAM, tumor-associated macrophage; Th, T helper cell; Treg, regulatory T cell; Cor, R value of Spearman’s correlation; None, correlation without adjustment; Purity; correlation adjusted by purity. ns, P ≥ 0.05; *P < 0.05; **P < 0.01; ***P < 0.001.
